# Treatment Strategies and Prognosis of Patients With Synchronous or Metachronous Colorectal Peritoneal Metastases: A Population-Based Study

**DOI:** 10.1245/s10434-021-10190-z

**Published:** 2021-06-02

**Authors:** C. Bakkers, R. J. Lurvink, A. Rijken, S. W. Nienhuijs, N. F. Kok, G. J. Creemers, C. Verhoef, V. E. Lemmens, F. N. van Erning, I. H. De Hingh

**Affiliations:** 1 Department of Surgery, Catharina Cancer Institute, P. O. Box 1350, 5602 ZA Eindhoven, Netherlands; 2 Department of Research, Netherlands Comprehensive Cancer Organization, Utrecht, Netherlands; 3 Department of Surgery, Netherlands Cancer Institute, Amsterdam, Netherlands; 4 Department of Medical Oncology, Catharina Cancer Institute, Eindhoven, Netherlands; 5 Department of Surgery, Erasmus Medical Center, Rotterdam, Netherlands; 6 GROW–School for Oncology and Developmental Biology, Maastricht University, Maastricht, Netherlands

## Abstract

**Background:**

This study aimed to compare treatment strategies and survival of patients with synchronous colorectal peritoneal metastases (CPM) and patients with metachronous CPM in a nationwide cohort.

**Methods:**

All patients from the Netherlands Cancer Registry with synchronous or metachronous CPM whose primary colorectal cancer (CRC) was diagnosed between 1 January and 30 June 2015 were included in the study. Treatments were categorized as (A) cytoreductive surgery and hyperthermic intraperitoneal chemotherapy [CRS-HIPEC]; (B) palliative treatment; or (C) best supportive care. Overall survival (OS) for all the patients and disease-free survival (DFS) for those who underwent CRS-HIPEC were compared between the two groups.

**Results:**

Of 7233 patients, 743 had a diagnosis of CPM, including 409 patients with synchronous CPM and 334 patients with metachronous CPM. The median OS was 8.1 months for the patients with synchronous CPM versus 12 months for the patients with metachronous CPM (*p* = 0.003). After multivariable correction, OS no longer differed between the patients with synchronous CPM and those with metachronous CPM (HR 1.03 [0.83–1.27])*.* The patients with metachronous CPM more often underwent CRS-HIPEC than the patients with synchronous CPM (16 % vs 8 %; *p* = 0.001). The two groups did not differ statistically in terms of DFS and OS (median DFS, 21.5 vs 14.1 months, respectively;* p* = 0.094; median OS, 37.8 vs. 35.8 months, respectively;* p* = 0.553).

**Conclusion:**

This population-based study showed that survival for the patients with synchronous CPM and patients with metachronous CPM did not significantly differ. This suggests that a similar prognosis may be expected for patients selected for treatment regardless of the onset of CPM.

**Supplementary Information:**

The online version contains supplementary material available at 10.1245/s10434-021-10190-z.

Colorectal cancer (CRC) is the third most common malignancy worldwide.^1^ Approximately one third of patients are confronted with metastatic disease, either at the time of diagnosis or later during follow-up evaluation after curative treatment.^2^ After the liver, the peritoneum is the second most common metastatic site of CRC.^3^,^4^ Colorectal peritoneal metastases (CPM), occurring in about 10 % of CRC patients, are diagnosed during the initial treatment of the primary tumor (synchronous peritoneal metastases) or during follow-up evaluation (metachronous peritoneal metastases).^2^

Although the risk factors for synchronous and metachronous CPM are alike,^2^ it is unknown whether the tumor behavior differs between synchronous peritoneal metastases and metachronous peritoneal metastases. A different tumor behavior may result in a different prognosis and therefore require adjusted treatment strategies.^5^,^6^

Recently, an Italian society of experts in peritoneal surface malignancies defined and approved different diagnostic and therapeutic algorithms for synchronous peritoneal metastases and metachronous peritoneal metastases.^7^ However, most international guidelines do not take the presentation of CPM into account in recommendations regarding treatment.^8^–^11^ Also, in some randomized trials, the synchronous or metachronous presentation of CPM is used as a stratification factor.^12^ Still, it remains unclear whether any differences exist between synchronous CPM and metachronous CPM and, if so, how this affects prognosis in an unselected population.

This population-based study aimed to provide insight into the treatment strategies and prognosis of patients with synchronous CPM and those with metachronous CPM and to identify characteristics associated with prognosis, providing an up-to-date basis for future clinical research investigating patients with synchronous CPM and those with metachronous CPM.

## Methods

### Data Source

Data from the Netherlands Cancer Registry (NCR) were used for the performance of the current nationwide population-based cohort study. The NCR registers all newly diagnosed malignancies in the Netherlands, and trained data managers from the NCR routinely collect patient, tumor, and treatment characteristics. Each year, the vital status of all patients is checked by linkage to the municipal administrative database, in which all deaths of Dutch inhabitants are registered.

For the current study, the latest linkage to the municipal administrative database was performed in February 2020. In 2019, all patients with a diagnosis of CRC determined between 1 January and 30 June 2015 were reassessed to obtain follow-up information on locoregional and/or systemic recurrences and their treatment. All data were rendered anonymous, obviating approval for the study by the medical ethics committee.

### Patients and Characteristics

The study excluded patients with an appendiceal tumor, a neuro-endocrine tumor, or a tumor with histology other than adenocarcinoma. For the analyses, the study included only patients who experienced synchronous or metachronous peritoneal metastases, defined as present in any of the following metastatic locations according to the International Classification of Disease–Oncology [ICD-O]: C16.0–C16.9, C17.0–C17.9, C18.0–C18.9, C19.9, C20.9, C21.8, C23.9, C26.9, C48.0–C48.8, C49.4–C49.5, C52.9, C53.9, C54.0–C54.9, C55.9, C56.9, C57.0–C57.8, C66.9, C67.0–C67.9, or C76.2). All metastases in other locations were registered as systemic metastases. Metastases were considered synchronous if diagnosed 90 days or less after surgery for primary CRC or 90 days or less after diagnosis if no surgery for primary CRC was performed. Among the patients without synchronous peritoneal metastases, only those who underwent surgery for primary CRC were evaluated for the development of metachronous metastases. Metastases were considered metachronous if diagnosed longer than 90 days after surgery for primary CRC.

The primary tumor location was subcategorized according to the ICD-O as (1) right-sided colon (C18.0, C18.2–18.4): cecum, ascending colon, hepatic flexure, transverse colon; (2) left-sided colon (C18.5–18.7): splenic flexure, descending colon and sigmoid; or (3) rectum (C19.9–20.9): rectosigmoid and rectum. The primary tumor histology was defined as adenocarcinoma (8000, 8010, 8020, 8140, 8144, 8210, 8211, 8220 8255, 8261, 8262, 8263, 8560), mucinous adenocarcinoma (8480, 8481), or signet ring cell carcinoma (8490).

The treatment of peritoneal metastases was defined as (1) cytoreductive surgery and hyperthermic intraperitoneal chemotherapy [CRS-HIPEC] with or without systemic chemotherapy and/or radiotherapy; (2) palliative treatment: systemic chemotherapy, metastasectomy, and/or radiotherapy without curative intent; or (3) no tumor-directed treatment, only best supportive care (BSC).

### Statistical Analyses

The baseline characteristics of the patients with synchronous peritoneal metastases were compared with those of patients with metachronous peritoneal metastases. Continuous variables are presented as mean ± standard deviation and were compared using the unpaired *t* test. Categorical variables are presented as number (%) and were compared with the chi-square test. Different treatment strategies between the patients with synchronous and those with metachronous peritoneal metastases were compared using the chi-square test. All the tests were two-sided, and a *p* value lower than 0.05 was considered statistically significant.

Median overall survival (OS) was determined with the Kaplan-Meier method and compared between the patients with synchronous CPM and those with metachronous CPM as well as between the patients treated with different treatment strategies using the log-rank test. The median OS was calculated from the first diagnosis of peritoneal metastases until death or loss to follow-up evaluation. Disease free survival (DFS) was determined only for the patients who underwent CRS-HIPEC and was calculated from the date of CRS-HIPEC until the diagnosis of metastases (locoregional and/or systemic metastases) thereafter.

Univariable Cox regression analyses were performed for the patients with peritoneal metastases (as one group) and for the patients with synchronous or metachronous peritoneal metastases (as two groups) to identify risk factors affecting OS. To prevent overfitting, variables with a *p* value lower than 0.10 were subsequently combined in multivariable Cox regression models with respect to the number of deaths in each group (10 deaths per degree of freedom). Dummy variables of missing data were included in the multivariable analyses. All analyses were performed using SAS statistical software (SAS system 9.4, SAS Institute, Cary, NC, USA).

## Results

### Study Population

Between 1 January and 30 June 2015, CRC was diagnosed for 7233 patients. Of these patients, 409 (5.7 %) presented with synchronous peritoneal metastases. During follow-up evaluation, metachronous peritoneal metastases was diagnosed for 334 (5.7 %) of 5860 patients without synchronous peritoneal metastases who underwent surgery for primary CRC. The median follow-up period after surgery was 38.4 months (interquartile range [IQR], 15.3–45.4 months). The baseline characteristics of the patients with synchronous CPM and those with metachronous CPM are presented in Table 1. Poorly differentiated or undifferentiated tumor, T4 tumor stage, and synchronous systemic metastases were more frequently diagnosed for the patients with synchronous peritoneal metastases than for those with metachronous peritoneal metastases.

**Table 1:** Baseline characteristics

	Synchronous PM (*n* = 409) *n* (%)	Metachronous PM (*n* = 334) *n* (%)	*p* value
Mean age (years)	69 ± 12	67 ± 11	0.062
**Sex**			
Male	225 (55)	185 (55)	0.918
Female	184 (45)	149 (45)	
ASA			
ASA 1	29 (7)	63 (19)	<0.001
ASA 2	138 (34)	165 (49)	
ASA ≥3	75 (18)	57 (17)	
Missing data^a^	167 (41)	49 (15)	
Primary tumor location			
Right-sided colon	186 (45)	126 (38)	<0.001
Left-sided colon	173 (42)	132 (39)	
Rectum	50 (12)	76 (23)	
Tumor differentiation			
Good/moderate	167 (41)	248 (74)	0.002
Poor/undifferentiated	67 (16)	52 (16)	
Missing data^a^	175 (43)	34 (10)	
Tumor histology			
Adenocarcinoma	313 (77)	282 (86)	0.001
Mucinous adenocarcinoma	65 (16)	35 (11)	
Signet ring cell carcinoma	31 (6)	9 (3)	
Tumor stage			
T0-3	118 (29)	210 (63)	<0.001
T4	192 (47)	124 (37)	
Missing data^a^	99 (24)	0 (0)	
Nodal stage			
N0	83 (20)	97 (29)	<0.001
N1	104 (25)	126 (38)	
N2	165 (40)	109 (32)	
Missing data^a^	57 (14)	2 (1)	
Synchronous systemic metastases			
No	166 (41)	252 (75)	<0.001
Yes	243 (59)	82 (25)	
Colon perforation			
No	203 (50)	293 (88)	0.106
Yes	24 (6)	21 (6)	
Missing data^a^	182 (44)	20 (6)	

PM, peritoneal metastases; ASA, American Society of Anesthesiologists

^a^Missing data were not included in the chi-square analyses.

### Treatments

**Fig. 1.**
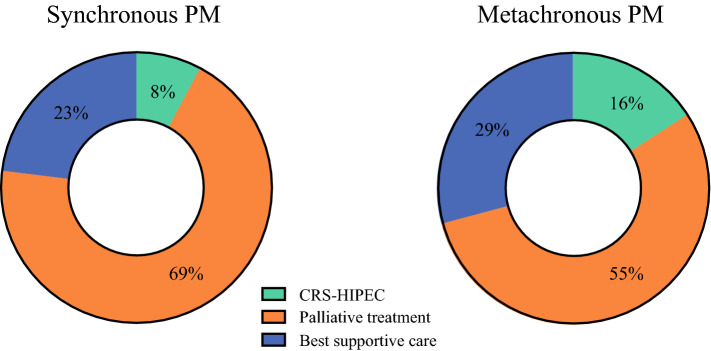
Treatment strategies for patients with synchronous or metachronous colorectal peritoneal metastases.

Figure 1 provides an overview of the treatment strategies applied for the patients with synchronous or metachronous peritoneal metastases. Overall, the distribution of applied treatment strategies differed significantly between patients with synchronous peritoneal metastases and those with metachronous peritoneal metastases (*p* < 0.001). The patients with metachronous peritoneal metastases more frequently underwent CRS-HIPEC (16 % vs 8 %; *p* = 0.001) and less frequently underwent palliative treatment (55 % vs 69 %; *p* < 0.001) than the patients with synchronous peritoneal metastases. The number of patients who received BSC was similar between the metachronous peritoneal metastases group and synchronous peritoneal metastases group (29 % vs 23 %; *p* = 0.051).

### Disease-Free Survival

**Fig. 2.**
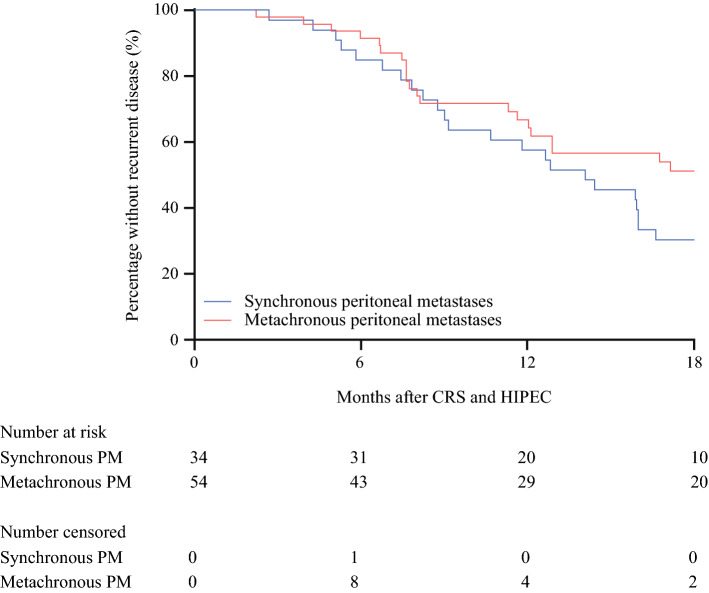
Disease-free survival of patients with synchronous or metachronous colorectal peritoneal metastases after cytoreductive surgery and hyperthermic intraperitoneal chemotherapy (CRS-HIPEC).

**Fig. 3.**
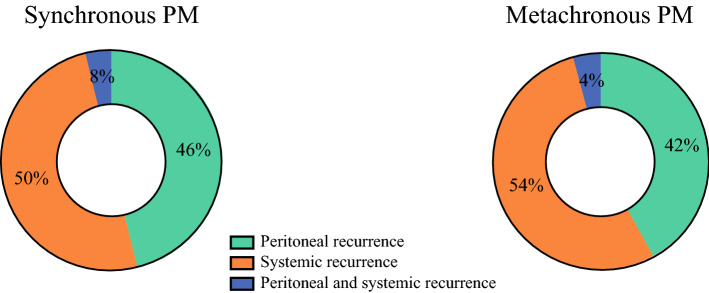
Sites of first recurrent disease after cytoreductive surgery and hyperthermic intraperitoneal chemotherapy (CRS-HIPEC) in patients with synchronous or metachronous colorectal peritoneal metastases.

The DFS of the patients with synchronous or metachronous peritoneal metastases who underwent CRS-HIPEC is shown in Fig. 2. The median DFS was 14.1 months (IQR, 8.2–29.2 months) for the patients with synchronous peritoneal metastases and 21.5 months (IQR, 8.0, not reached) for the patients with metachronous peritoneal metastases, but the difference was not significant *(p* = 0.094). The site or sites of first recurrence after CRS-HIPEC in the patients with synchronous or metachronous peritoneal metastases are shown in Fig. 3. No differences in the pattern of recurrence were observed between the patients with synchronous peritoneal metastases and those with metachronous peritoneal metastases (*p* = 0.950).

### Overall Survival

**Fig. 4.**
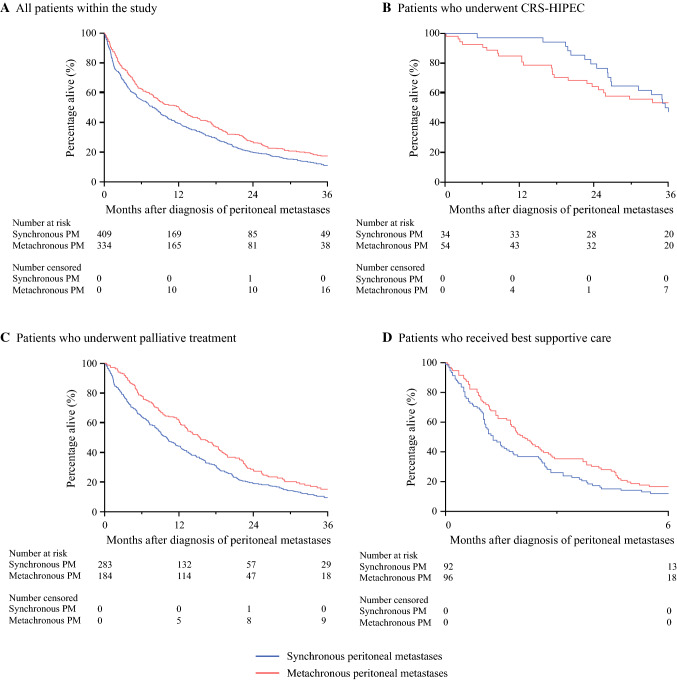
Overall survival of patients with synchronous or metachronous colorectal peritoneal metastases. **a** All the patients in the study. **b** All the patients who underwent cytoreductive surgery and hyperthermic intraperitoneal chemotherapy (CRS-HIPEC). **c** All the patients who underwent palliative treatment. **d** All the patients who received best supportive care**.**

The median OS of all the patients with peritoneal metastases was 9.1 months (IQR, 2.6–22.5 months), with a 1-year OS of 44 % and a 3-year OS of 13 %. The patients with synchronous peritoneal metastases had a worse OS (median, 8.1 months; IQR, 2.0–20.5 months) than the patients with metachronous peritoneal metastases (12.0 months; IQR, 3.5–25.5 months) (*p* = 0.003; Fig. 4a)*.* However, after multivariable Cox regression analysis, the presentation of peritoneal metastases did not affect OS significantly (metachronous peritoneal metastases vs synchronous peritoneal metastases: HR, 1.03; 95 % CI 0.83–1.27) (Table 2).

**Table 2:** Uni- and multivariable Cox regression analyses for overall survival of the entire study cohort

	Median OS (months)	Univariable analyses			Multivariable analyses		
		HR	95 % CI	*p* value	HR	95 % CI	*p* value
Age (years)				**<0.001**			
<50	15.6	0.80	0.59–1.09		0.84	0.61–1.15	0.283
50–74	11.9	Ref	Ref		Ref	Ref	Ref
≥75	4.2	1.74	1.47–2.07		1.05	0.86–1.26	0.654
Sex				0.156			
Male	9.4	Ref	Ref		–	–	–
Female	8.7	1.12	0.96–1.31		–	–	–
ASA score				**<0.001**			
ASA 1	17.6	0.72	0.55–0.94		0.92	0.69–1.22	0.571
ASA 2	12.1	Ref	Ref		Ref	Ref	Ref
ASA 3–6	5.7	1.44	1.16–1.79		1.16	0.93–1.45	0.202
Missing data	4.6	1.80	1.50–2.17		1.26	1.02–1.56	0.034
Primary tumor location				**0.019**			
Right colon	7.3	Ref	Ref		Ref	Ref	Ref
Left colon	11.4	0.79	0.66–0.93		0.87	0.73–1.05	0.141
Rectum	9.7	0.87	0.69–1.08		1.12	0.88–1.44	0.356
Primary tumor differentiation				**<0.001**			
Good/moderate	14.2	Ref	Ref		Ref	Ref	Ref
Poor/undifferentiated	3.6	2.52	2.03–3.13		**2.00**	**1.57–2.52**	**<0.001**
Missing data	5.3	1.66	1.39–1.98		1.21	0.97–1.51	0.096
Tumor histology				**0.003**			
Adenocarcinoma	9.5	Ref	Ref		Ref	Ref	Ref
Mucinous adenocarcinoma	9.1	0.97	0.77–1.22		0.83	0.64–1.07	0.148
Signet ring cell carcinoma	3.8	1.83	1.32–2.53		**1.51**	**1.06–2.15**	**0.024**
Tumor stage				**<0.001**			
T0-3	9.5	Ref	Ref		Ref	Ref	Ref
T4	11.7	1.02	0.86–1.21		1.12	0.92–1.35	0.257
Missing data	3.7	2.21	1.75–2.79		1.20	0.90–1.60	0.220
Nodal stage				**<0.001**			
N0	12.8	0.74	0.60–0.91		0.82	0.66–1.03	0.087
N1	10.8	0.91	0.75–1.10		1.01	0.83–1.23	0.937
N2	8.3	Ref	Ref		Ref	Ref	Ref
Missing data	2.8	2.28	1.71–3.04		1.74	1.27–2.38	<0.001
Synchronous systemic metastases				**<0.001**			
No	12.1	Ref	Ref		Ref	Ref	Ref
Yes	6.7	1.36	1.17–1.59		**1.22**	**1.02–1.47**	**0.034**
Tumor perforation				**<0.001**			
No	12.2	Ref	Ref		Ref	Ref	Ref
Yes	9.4	0.98	0.70–1.38		0.99	0.69–1.41	0.958
Missing data	8.2	1.85	1.55–2.19		1.06	0.86–1.31	0.593
Presentation of PM				**0.003**			
Synchronous	8.1	Ref	Ref		Ref	Ref	Ref
Metachronous	12.0	0.79	0.67–0.92		1.03	0.83–1.27	0.813
Treatment of PM				**<0.001**			
Best supportive care	1.8	4.56	3.77–5.51		**4.44**	**3.57–5.52**	**<0.001**
Palliative treatment	12.2	Ref	Ref		Ref	Ref	Ref
CRS-HIPEC	36.0	0.31	0.23–0.43		**0.40**	**0.29–0.55**	**<0.001**

OS, overall survival; HR, hazard ratio; CI, confidence interval; ASA, American Society of Anesthesiologists; PM, peritoneal metastases; CRS, cytoreductive surgery; HIPEC, hyperthermic intraperitoneal chemotherapy

For the patients who underwent CRS-HIPEC, the median OS of the entire cohort was 36 months (IQR, 22.5 months, not reached). The median OS did not differ significantly between the patients with synchronous peritoneal metastases (35.8 months; IQR, 26.2 months, not reached) and those with metachronous peritoneal metastases (37.8 months; IQR, 17.3 months, not reached) (*p* = 0.553; Fig. 4b).

For the patients who received palliative treatment, the median OS of the entire cohort was 12.2 months (IQR, 4.9–22.7 months). The OS was worse for the patients with synchronous peritoneal metastases (median, 10.0 months; IQR 3.6–20.6 months) than for those with metachronous peritoneal metastases (15.4 months; IQR, 6.8–25.6 months; *p* < 0.001 (Fig. 4c)*.*

For the patients who received only BSC, the median OS of the entire cohort was 1.8 months (IQR, 0.9–3.9 months). The OS was worse for the patients with synchronous peritoneal metastases (1.3 months; IQR, 0.6–3.2 months) than for the patients with metachronous peritoneal metastases (2.1 months; IQR, 1.0–4.6 months; *p* = 0.021 (Fig. 4d)*.*

### Factors Associated With Overall Survival

The results of the univariable Cox regression analyses for the OS of the patients with synchronous and those with metachronous peritoneal metastases are shown in Table S1. The results of the multivariable Cox regression analyses of the patients with synchronous and those with metachronous peritoneal metastases are shown in Table 3.

**Table 3:** Multivariable Cox regression analyses for overall survival of patients with synchronous and metachronous peritoneal metastases

	Synchronous PM				Metachronous PM			
	Median OS (months)	HR	95 % CI	*p* value	Median OS (months)	HR	95 % CI	*p* value
Age (years)								
<50	15.9	0.86	0.56–1.32	0.494	14.5	0.84	0.52–1.35	0.472
50–74	10.0	Ref	Ref	Ref	14.3	Ref	Ref	Ref
≥75	3.8	0.95	0.75–1.21	0.672	4.6	1.19	0.86–1.64	0.294
Sex								
Male	8.4	–	–	–	12.2	Ref	Ref	Ref
Female	7.3	–	–	–	10.4	1.01	0.78–1.30	0.969
ASA stage								
ASA 1	18.6	0.88	0.55–1.38	0.567	16.4	0.95	0.65–1.38	0.775
ASA 2	11.3	Ref	Ref	Ref	12.2	Ref	Ref	Ref
ASA ≥3	5.2	**1.43**	**1.05–1.94**	**0.023**	6.1	0.87	0.62–1.23	0.420
Missing data	3.8	1.31	1.01–1.72	0.049	10.4	1.15	0.80–1.67	0.457
Primary tumor location								
Right-sided colon	7.0	Ref	Ref	Ref	8.5	–	–	–
Left-sided colon	9.9	0.93	0.74–1.17	0.545	13.5	–	–	–
Rectum	6.3	**1.63**	**1.15–2.31**	**0.006**	12.4	–	–	–
Primary tumor differentiation								
Good/moderate	14.9	Ref	Ref	Ref	14.1	Ref	Ref	Ref
Poor/none	3.7	**2.06**	**1.48–2.85**	**<0.001**	3.2	**2.00**	**1.42–2.80**	**<0.001**
Missing data	5.3	1.23	0.94–1.59	0.130	12.1	0.75	0.43–1.30	0.301
Tumor histology								
Adenocarcinoma	8.2	Ref	Ref	Ref	13.0	Ref	Ref	Ref
Mucinous adenocarcinoma	9.9	0.81	0.59–1.11	0.191	6.6	1.22	0.74–2.01	0.434
Signet ring cell carcinoma	4.2	1.32	0.87–2.01	0.192	3.2	**2.70**	**1.24–5.88**	**0.012**
Tumor stage								
T0-3	7.8	Ref	Ref	Ref	12.4	–	–	–
T4	12.0	1.19	0.91–1.56	0.203	11.2	–	–	–
Missing data	3.7	1.25	0.91–1.71	0.163		–	–	–
Nodal stage								
N0	8.4	0.96	0.71–1.31	0.802	17.9	**0.64**	**0.45–0.92**	**0.015**
N1	9.0	0.97	0.73–1.28	0.812	12.2	1.08	0.80–1.46	0.622
N2	10.9	Ref	Ref	Ref	5.3	Ref	Ref	Ref
Missing data	2.8	1.70	1.21–2.39	0.002	10.5	2.06	0.49–8.64	0.323
Synchronous systemic metastases								
No	10.6	Ref	Ref	Ref	12.7	–	–	–
Yes	5.5	1.18	0.93–1.49	0.174	8.7	–	–	–
Tumor perforation								
No	12.6	Ref	Ref	Ref	12.0	–	–	–
Yes	13.3	0.80	0.49–1.30	0.366	7.6	–	–	–
Missing data	7.0	1.24	0.96–1.59	0.100	13.1	–	–	–
Adjuvant treatment after primary surgery for colorectal cancer								
No	NA	NA	NA	NA	9.0	Ref	Ref	Ref
Yes	NA	NA	NA	NA	17.4	0.84	0.63–1.13	0.250
Treatment of PM								
Best supportive care	1.3	**4.11**	**3.00–5.63**	**<0.001**	2.1	**4.95**	**3.60–6.81**	**<0.001**
Palliative treatment	10.0	Ref	Ref	Ref	15.4	Ref	Ref	Ref
CRS- HIPEC	35.8	**0.35**	**0.22–0.57**	**<0.001**	37.8	**0.43**	**0.28–0.67**	**<0.001**

OS, overall survival; HR, adjusted hazard ratio; CI, confidence interval; ASA, American Society of Anesthesiologists; NA, not applicable; PM, peritoneal metastases; CRS, cytoreductive surgery; HIPEC, hyperthermic intraperitoneal chemotherapy

For the patients with synchronous peritoneal metastases, a worse OS was significantly associated with an American Society of Anesthesiology (ASA) score of 3 or higher (adjusted HR 1.43; 95 % confidence interval [CI], 1.05–1.94), a primary tumor located in the rectum (HR 1.63; 95 % CI 1.15–2.31]), and a poorly differentiated or undifferentiated primary tumor (HR 2.06; 95 % CI 1.48–2.85). Treatment with CRS-HIPEC was significantly associated with better OS than palliative treatment (HR 0.35; 95 % CI 0.22–0.57). Best supportive care was significantly associated with worse OS than palliative treatment (HR 4.11; 95 % CI 3.00–5.63).

For the patients with metachronous peritoneal metastases, a worse OS was significantly associated with a poorly differentiated or undifferentiated primary tumor (HR 2.00; 95 % CI 1.42–2.80) and signet ring cell carcinoma (HR 2.70; 95 % CI 1.24–5.88). An N0 status was significantly associated with a better OS (HR 0.64; 95 % CI 0.45–0.92). Treatment with CRS-HIPEC was significantly associated with a better OS than palliative treatment (HR 0.43; 95 % CI 0.28–0.67). Best supportive care was significantly associated with a worse OS than palliative treatment (HR 4.95; 95 % CI 3.60–6.81).

## Discussion

To the best of our knowledge, the current study was the first to compare treatment strategies and survival between patients with synchronous CPM and those with metachronous CPM in a nationwide cohort. Overall survival did not differ significantly between the patients with synchronous peritoneal metastases and those with metachronous peritoneal metastases after correction for covariables, although the patients with metachronous peritoneal metastases were more often treated with CRS-HIPEC than the patients with synchronous peritoneal metastases. Moreover, neither OS nor DFS differed significantly the between patients with synchronous peritoneal metastases and those with metachronous peritoneal metastases who underwent CRS-HIPEC.

Although the OS for the patients with synchronous peritoneal metastases and those with metachronous peritoneal metastases did not differ significantly after correction for covariables, the more favorable crude OS of the patients with metachronous peritoneal metastases could be explained in different ways. First, the late presentation of metachronous peritoneal metastases may itself suggest a less aggressive tumor behavior, thus resulting in better OS. However, metachronous peritoneal metastases occur primarily in patients with high-risk tumors, who are designated for adjuvant systemic therapy after primary surgery according to most national and international guidelines to minimize the risk of metastatic recurrence.^10^,^13^ Therefore, if metachronous peritoneal metastases occur regardless of adjuvant systemic therapy, it may instead suggest a more aggressive tumor biology.

Second, the better crude OS for patients with metachronous peritoneal metastases may have been related to lead-time bias. After primary treatment for CRC, these patients underwent standardized follow-up evaluation for several years, which may have resulted in the early diagnosis of less advanced metachronous peritoneal metastases. On the other hand, the patients with synchronous peritoneal metastases may have remained unnoticed until an advanced stage of disease given the absence of clinical symptoms in most of these patients.^14^ The higher number of patients with metachronous peritoneal metastases treated with CRS-HIPEC compared with the number of patients who had synchronous peritoneal metastases treated with CRS-HIPEC in the current study supports this hypothesis. Furthermore, synchronous CPMs are frequently discovered (in an emergency setting) in non-academic hospitals that are not specialized HIPEC centers, which is known to affect the likelihood of a patient eventually undergoing CRS-HIPEC.^15^

In the current study, DFS and OS for the patients with synchronous peritoneal metastases and those with metachronous peritoneal metastases who underwent CRS-HIPEC were nonsignificantly different. Another comparative study, which included patients from two Dutch HIPEC centers, showed a significantly longer DFS (15 months) for 231 patients with synchronous peritoneal metastases compared with 11 months for 202 patients who had metachronous peritoneal metastases, without a difference in OS.^16^ Recently, Min Wong et al.^5^ demonstrated no differences in DFS, but showed a better OS for patients with metachronous peritoneal metastases (45 months) than for patients with synchronous peritoneal metastases (27 months). A similar trend was observed in a third study, with no difference in DFS but a better OS for patients with metachronous peritoneal metastases (28 months) than for patients with synchronous peritoneal metastases (7 months). However, in the latter study, survival was calculated from the diagnosis of primary CRC instead of from the diagnosis of peritoneal metastases, explaining the much longer survival of patients with metachronous CPM.^6^

Other population-based studies that reported the survival of all patients with synchronous peritoneal metastases demonstrated a median OS of 8 to 9 months (diagnosis in 2002–2011),^4^,^17^ similar to that of the current study. For metachronous peritoneal metastases, a median OS of 6 months was reported (diagnosis of primary CRC in 2003–2008),^18^ which is lower than the OS for the patients in the current study. This improvement over time for patients with metachronous peritoneal metastases may be due to improved diagnostic methods and better follow-up evaluation, with higher awareness for metachronous peritoneal metastases after primary surgery for CRC, especially because no improvement was found in patients with synchronous peritoneal metastases. However, currently available data on the association between the intensity of follow-up evaluation after primary CRC treatment and OS is rather contradictory to two meta-analyses conducted in 2019^19^ and 2016^20^ concluding that the intensified surveillance of CRC patients does not result in a cancer-specific survival benefit. Furthermore, a systematic review from 2017 concluded that although patients with stages 1 to 3 CRC may experience a survival benefit, the existence of this benefit is questionable for patients with stage 4 CRC.^21^ In addition, a randomized controlled trial concluded that intensified carcinoembryonic antigen (CEA) measurements resulted in earlier recurrence detection and a higher proportion of patients who could be treated with curative intent. However, this did not result in a survival benefit.^22^,^23^ As previously noted, patients with synchronous CPM were less likely to be treated with CRS-HIPEC, which may be responsible for this phenomenon.

The extent of peritoneal metastases (peritoneal cancer index [PCI]) was unknown for the patients included in the current study because this is not registered by the NCR. The PCI is known to affect prognosis^24^ and, hypothetically, the patients with metachronous peritoneal metastases may have had a lower PCI than the patients with synchronous peritoneal metastases, explaining the higher percentage of patients who had metachronous CPM treated with CRS-HIPEC. However, such a difference was not observed in previous studies.^5^,^16^ Furthermore, the primary tumor being *in situ* in patients with synchronous peritoneal metastases may have had a negative impact on treatment and prognosis. Moreover, some patients with metachronous peritoneal metastases were excluded from this analysis if they had not undergone surgery for primary CRC. These patients may have had a worse prognosis because they were not able to undergo surgery. The exclusion of patients who did not undergo surgery may have led to an overestimation of the OS in the group of patients with metachronous peritoneal metastases. Also, selection bias likely will have influenced the received treatment, possibly resulting in overestimation of the beneficial effect of CRS-HIPEC and, to a lesser extent, palliative treatment because patients who are fit enough to receive treatment are more likely to actually undergo treatment than patients with a poor clinical condition. Unfortunately, the NCR does not register the reason for the choosing or not choosing of a certain treatment. Still, all patients were treated according to the national guideline for CRC, which defines the selection criteria for eligibility to receive CRS-HIPEC (e.g., PCI <20, limited small bowel involvement, absence of systemic metastases).

In conclusion, the OS did not differ significantly between the synchronous CPM and metachronous CPM patients. Also, within the subgroup of patients treated with CRS-HIPEC, DFS and OS as well as the pattern of recurrence were comparable. This suggests that a similar prognosis may be expected for patients selected to undergo treatment regardless of the onset of CPM.

## Supplementary Information

Below is the link to the electronic supplementary material.

Supplementary file1 (DOCX 24 kb)
