# Supplementary material for: Treatment Strategies and Prognosis of Patients With Synchronous or Metachronous Colorectal Peritoneal Metastases: A Population-Based Study
Source: Ann Surg Oncol. 2021 Jun 2;28(13):9073–83. doi: 10.1245/s10434-021-10190-z (PMC8591028; doi:10.1245/s10434-021-10190-z)
Supplement: Supplementary file 1 — Supplementary file1 (DOCX 24 kb) [file 10434_2021_10190_MOESM1_ESM.docx]

**Supplementary table 1. Univariable Cox Regression analyses for overall survival in patients with synchronous and metachronous peritoneal metastases.**

|  | **Synchronous PM** | | | | **Metachronous PM** | | | |
| --- | --- | --- | --- | --- | --- | --- | --- | --- |
|  | **Median OS (months)** | **HR** | **95% CI** | **P value** | **Median OS (months)** | **HR** | **95% CI** | **P value** |
| **Age** |  |  |  | **<0.001** |  |  |  | **<0.001** |
| <50 | 15.9 | 0.74 | 0.48-1.14 |  | 14.5 | 0.86 | 0.54-1.36 |  |
| 50-74 | 10.0 | Ref | Ref |  | 14.3 | Ref | Ref |  |
| ≥75 | 3.8 | 1.57 | 1.27-1.95 |  | 4.6 | 1.95 | 1.48-2.56 |  |
| **Sex** |  |  |  | 0.728 |  |  |  | **0.081** |
| Male | 8.4 | Ref | Ref |  | 12.2 | Ref | Ref |  |
| Female | 7.3 | 1.04 | 0.85-1.27 |  | 10.4 | 1.24 | 0.97-1.58 |  |
| **ASA score** |  |  |  | **<0.001** |  |  |  | **0.002** |
| ASA 1 | 18.6 | 0.76 | 0.49-1.17 |  | 16.4 | 0.70 | 0.49-0.99 |  |
| ASA 2 | 11.3 | Ref | Ref |  | 12.2 | Ref | Ref |  |
| ASA ≥3 | 5.2 | 1.42 | 1.06-1.91 |  | 6.1 | 1.48 | 1.07-2.04 |  |
| Missing data | 3.8 | 2.04 | 1.60-2.59 |  | 10.4 | 1.26 | 0.89-1.80 |  |
| **Primary tumor location** |  |  |  | **0.040** |  |  |  | 0.162 |
| Right-sided colon | 7.0 | Ref | Ref |  | 8.5 | Ref | Ref |  |
| Left-sided colon | 9.9 | 0.79 | 0.64-0.98 |  | 13.5 | 0.78 | 0.59-1.03 |  |
| Rectum | 6.3 | 1.11 | 0.81-1.53 |  | 12.4 | 0.79 | 0.57-1.09 |  |
| **Primary tumor differentiation** |  |  |  | **<0.001** |  |  |  | **<0.001** |
| Good/moderate | 14.9 | Ref | Ref |  | 14.1 | Ref | Ref |  |
| Poor/none | 3.7 | 2.67 | 1.98-3.61 |  | 3.2 | 2.34 | 1.70-3.24 |  |
| Missing data | 5.3 | 1.82 | 1.45-2.28 |  | 12.1 | 1.09 | 0.73-1.65 |  |
| **Tumor histology** |  |  |  | **0.064** |  |  |  | **0.006** |
| Adenocarcinoma | 8.2 | Ref | Ref |  | 13.0 | Ref | Ref |  |
| Mucinous adenocarcinoma | 9.9 | 0.83 | 0.63-1.10 |  | 6.6 | 1.18 | 0.80-1.75 |  |
| Signet ring cell carcinoma | 4.2 | 1.43 | 0.98-2.07 |  | 3.2 | 3.62 | 1.84-7.11 |  |
| **Tumor stage** |  |  |  | **<0.001** |  |  |  | 0.552 |
| T0-3 | 7.8 | Ref | Ref |  | 12.4 | Ref | Ref |  |
| T4 | 12.0 | 0.92 | 0.72-1.17 |  | 11.2 | 1.08 | 0.84-1.38 |  |
| Missing data | 3.7 | 1.98 | 1.49-2.63 |  | - | - | - |  |
| **Nodal stage** |  |  |  | **<0.001** |  |  |  | **0.014** |
| N0 | 8.4 | 0.91 | 0.69-1.20 |  | 17.9 | 0.60 | 0.44-0.82 |  |
| N1 | 9.0 | 1.03 | 0.80-1.33 |  | 12.2 | 0.79 | 0.60-1.05 |  |
| N2 | 10.9 | Ref | Ref |  | 5.3 | Ref | Ref |  |
| Missing data | 2.8 | 2.38 | 1.75-3.26 |  | 10.5 | 1.24 | 0.31-5.05 |  |
| **Synchronous systemic metastases** |  |  |  | **0.005** |  |  |  | 0.187 |
| No | 10.6 | Ref | Ref |  | 12.7 | Ref | Ref |  |
| Yes | 5.5 | 1.34 | 1.09-1.65 |  | 8.7 | 1.21 | 0.92-1.59 |  |
| **Tumor perforation** |  |  |  | **<0.001** |  |  |  | 0.409 |
| No | 12.6 | Ref | Ref |  | 12.0 | Ref | Ref |  |
| Yes | 13.3 | 0.93 | 0.59-1.48 |  | 7.6 | 1.06 | 0.64-1.76 |  |
| Missing data | 7.0 | 2.19 | 1.77-2.72 |  | 13.1 | 0.69 | 0.39-1.24 |  |
| **Adjuvant treatment after surgery for primary colorectal cancer** |  |  |  |  |  |  |  | **0.042** |
| No | - | - | - |  | 9.0 | Ref | Ref |  |
| Yes | - | - | - |  | 17.4 | 0.77 | 0.59-0.99 |  |
| **Treatment of PM** |  |  |  | **<0.001** |  |  |  | **<0.001** |
| Best supportive care | 1.3 | 4.76 | 3.66-6.20 |  | 2.1 | 4.93 | 3.73-6.53 |  |
| Palliative treatment | 10.0 | Ref | Ref |  | 15.4 | Ref | Ref |  |
| CRS-HIPEC | 35.8 | 0.27 | 0.17-0.42 |  | 37.8 | 0.40 | 0.26-0.61 |  |
| *ASA* American association of anaesthesiologists; *CI* confidence interval; *CRS* cytoreductive surgery; *HIPEC* hyperthermic intraperitoneal chemotherapy; *HR* adjusted hazard ratio; *OS* overall survival; *PM* peritoneal metastases. | | | | | | | | |
